# Global study of holistic morphological effectors in the budding yeast *Saccharomyces cerevisiae*

**DOI:** 10.1186/s12864-018-4526-z

**Published:** 2018-02-20

**Authors:** Godai Suzuki, Yang Wang, Karen Kubo, Eri Hirata, Shinsuke Ohnuki, Yoshikazu Ohya

**Affiliations:** 1 Department of Integrated Biosciences, Graduate School of Frontier Sciences, University of Tokyo, Bldg. FSB-101, 5-1-5 Kashiwanoha, Kashiwa, Chiba Prefecture 277-8562 Japan; 2 AIST-UTokyo Advanced Operando-Measurement Technology Open Innovation Laboratory (OPERANDO-OIL), National Institute of Advanced Industrial Science and Technology (AIST), Bldg. Kashiwa Research Complex 2, 5-1-5 Kahiwanoha, Kashiwa, Chiba Prefecture 277-8565 Japan

**Keywords:** Yeast, *Saccharomyces cerevisiae*, Morphology, CalMorph, Phenotyping

## Abstract

**Background:**

The size of the phenotypic effect of a gene has been thoroughly investigated in terms of fitness and specific morphological traits in the budding yeast *Saccharomyces cerevisiae*, but little is known about gross morphological abnormalities.

**Results:**

We identified 1126 holistic morphological effectors that cause severe gross morphological abnormality when deleted, and 2241 specific morphological effectors with weak holistic effects but distinctive effects on yeast morphology. Holistic effectors fell into many gene function categories and acted as network hubs, affecting a large number of morphological traits, interacting with a large number of genes, and facilitating high protein expression. Holistic morphological abnormality was useful for estimating the importance of a gene to morphology. The contribution of gene importance to fitness and morphology could be used to efficiently classify genes into functional groups.

**Conclusion:**

Holistic morphological abnormality can be used as a reproducible and reliable gene feature for high-dimensional morphological phenotyping. It can be used in many functional genomic applications.

**Electronic supplementary material:**

The online version of this article (10.1186/s12864-018-4526-z) contains supplementary material, which is available to authorized users.

## Background

A central goal of genetics is to understand the relationship between genotype and phenotype. However, simple one-to-one mapping between genes and phenotypes is not easy for a number of reasons. Numerous complex genetic interactions occur in organisms, both among various genes and with the environment [1]. The phenotypes of living organisms are highly complex [2–4], and high-dimensional quantitative approaches have been applied to them considerably over the last few decades. Except in relatively simple cases, we know little about the precise extent to which mutations affect the activities and dynamics of cellular networks or the robustness of the cellular system [5].

Quantitative genetics, the statistical analysis of genetic effects on phenotypic variation, is a powerful approach that provides clues to elucidate the relationship between genotype and phenotype [6, 7]. Quantitative genetics have been investigated in the budding yeast *Saccharomyces cerevisiae* using natural yeast isolates [8, 9] with genotyping and phenotyping methods. The yeast gene deletion collection also made important contributions to our understanding of the biological functions of genes [10]. Growth phenotype [11, 12] and competitive fitness [10, 13] have been widely used in quantitative assays. Among the 5916 genes in the yeast genome, deletions of 18.7 and 15% of genes resulted in no growth and reduced fitness and growth, respectively, in rich YPD medium. Studies of fitness in gene deletion mutants uncovered phenotypic strength as a key gene feature. Hub genes in the genetic interaction network caused strong fitness defects when deleted [14–16]. Deletion of a gene that did not lead to expression of any gene product resulted in no phenotypic change [17]. Single mutations of redundant genes led to relatively weak fitness changes [18]. Therefore, the effect of phenotypic strength on fitness involves several effectors at various levels of phenotypic causality [19].

When yeast is observed under a microscope, it can be described morphologically from many points of view [20]. Morphology is one of the basic phenotypic characteristics of cells, and therefore conveys rich information about genetics. As a result, a greater number of genes affect yeast morphology than growth. More than half of non-essential deletion mutants exhibit morphological defects [21]. Thus, to create a complete functional wiring diagram of the yeast cell, a comprehensive understanding of gene functions and genetic interaction networks will be required, which must be based on extensive analysis of yeast morphology [21].

**Fig. 1:**
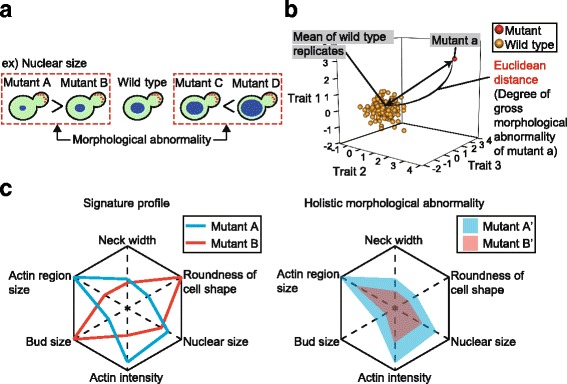
Degree of morphological abnormality. **a** Schematic representation of morphological abnormality (i.e., nucleus size) in budding yeast cells. Red and blue circles indicate the actin patch and nucleus, respectively. Inequality between mutants indicates a difference in the degree of morphological abnormality. **b** Schematic representation of holistic morphological abnormality in mutants. For each mutant, Euclidean distance from the mean of wild-type replicates is calculated in orthogonal phenotypic space to determine the degree of gross morphological abnormality. As an example, calculation of the Euclidean distance of mutant “a” in three-dimensional phenotypic space is shown. Red and orange spheres indicate mutant “a” and wild-type replicates, respectively. **c** Schematic representation of signature profiles and holistic morphological abnormalities in yeast morphological mutants. As an example, abnormalities of six morphological traits in a mutant from wild type are shown in spider charts. A center of a chart indicates no abnormality. In the left panel, red and blue lines indicate signature profiles of mutant A and B, respectively. In the right panel, red and blue areas indicate holistic morphological abnormalities of mutant A’ and B’, respectively. Sizes of the colored areas are proportional to degrees of the holistic morphological abnormalities

High-dimensional morphological analyses of cell shape, nuclear morphology, and actin morphology revealed yeast “morphology” mutants with distinct morphological traits compared to wild-type replicates [21, 22]. Genes that cause morphological abnormalities in a specific trait when deleted are considered important genes for that specific morphology [21, 22]. The magnitude of the specific morphological effect can be defined based on the phenotypic strength or phenotypic effect size [23] for each morphological trait (i.e., nuclear size, Fig. 1a). In contrast, some mutations have a high phenotypic strength or total effect size for many/all measured morphological parameters; use of Euclidian distance in morphological phenotypic space (Fig. 1b) allows us to define these mutations [23], which broadly affect the whole morphology of the organism, as causing “holistic morphological abnormalities”. As such, a holistic morphological abnormality can be defined based on high phenotypic strength or total effect size on morphology in multiple traits that are not intimately connected (Fig. 1b). It is independent of morphological signature [21, 22] (Fig. 1c, left), but rather reflects the total extent of the morphological defect (Fig. 1c, right). Holistic morphological effectors are defined here as non-essential genes with significant holistic effects on morphology. Although fitness has been used to estimate the magnitude of genetic effects, little is known about the holistic morphological effects of the genes.

This study was undertaken to elucidate holistic morphological effectors in budding yeast by comparing them with genes related to fitness. Holistic morphological abnormality was estimated in each non-essential deletion mutant by calculating the Euclidean distance between each mutant and the average of wild-type replicates in orthogonal morphological space. We found that holistic morphological effectors play important roles as intracellular network hubs. We also revealed that holistic morphological abnormality has only a weak correlation with fitness, suggesting that it can provide another ruler for measuring gene importance. Holistic morphological abnormality and fitness can be used to efficiently classify genes into functional categories. We propose a number of applications for holistic morphological abnormality in functional genomics.

## Results

### Calculation of holistic morphological abnormality

To study yeast non-essential deletion mutants with holistic morphological abnormalities, we employed yeast morphological data that was published previously [21]. The dataset analyzed contains 501 morphological traits, with 109 replicates of the wild-type strain and a single replicate each from 4718 non-essential gene deletion mutant strains. These 501 morphological traits are composed of 220 mean, 220 variance, and 61 ratio parameters regarding cell shape, actin, and nuclear DNA morphology.

To determine the degree of gross morphological abnormality in each mutant, we calculated the Euclidean distance between each mutant and the average of wild-type replicates in orthogonal phenotypic space after dimensional reduction (Fig. 1b). Dimensional reduction was carried out via principal component analysis (PCA) after normalization of morphological data. An advantage of using degenerate orthogonal space is that one can exclude bias caused by intrinsic correlations among the morphological parameters and eliminate principal components (PCs) with high experimental noise. We compressed the 501-dimensional morphological data for the 4718 mutants into 57 PCs, reaching 80% cumulative contribution ratio (CCR) (Additional file 1: Figure S1A). Lower variance of the wild type compared to mutants was almost assured with 57 PCs (Additional file 1: Figure S1B, C). The Euclidean distance between each mutant and the mean value of the wild type was then calculated with the 57 PC scores after standardization with wild-type replicates (Additional file 2: Table S1).

**Fig. 2:**
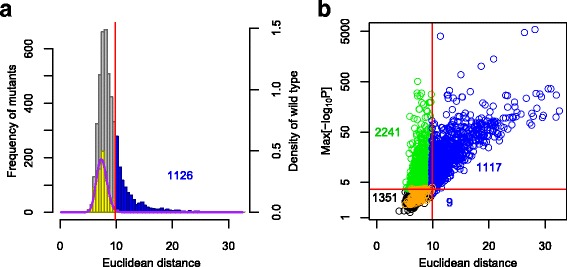
Identification of genes with holistic effects on yeast morphology. **a** Distribution of Euclidian distances (Additional file 2: Table S1). Blue, gray, and yellow boxes indicate non-essential gene deletion mutants with significant holistic morphological abnormality (left axis), other deletion mutants (left axis), and 109 replicates of the wild type (right axis), respectively, in 57-dimensional orthogonal space. The vertical solid red line indicates false discovery rate (FDR) = 0.01, and the purple curved line indicates a gamma distribution fitted to the wild-type replicates. **b** Scatter plot of non-essential gene deletion mutants in terms of holistic morphological abnormality (x-axis) and specific morphological abnormality (y-axis). The specific effect (y-axis) was defined as the maximum negative value of log-transformed *p* values for each of the 501 traits (Additional file 2: Table S1). Horizontal and vertical solid red lines indicate FDR = 0.01. Blue, green, orange, and black circles indicate 1126 holistic morphological mutants, 2241 specific morphological mutants, 109 replicates of the wild type, and 1351 other mutants, respectively

Comparing the distribution of Euclidean distances revealed that the distribution of the 4718 mutants was much broader than that of the 109 wild-type replicates (Fig. 2a). The Euclidean distance distribution of the mutants exhibited a long tail to the right, indicating that substantial gross morphological abnormality was caused by gene deletion from the genome.

### Holistic and specific effectors on yeast morphology

To identify genes with significant holistic effects on yeast morphology, a probability distribution of the wild-type replicates was estimated by fitting a gamma distribution, which was compared with each mutant. Of the 4718 non-essential genes, 1126 genes were identified as “holistic morphological effectors” at a false discovery rate (FDR) = 0.01 (blue in Fig. 2b). On the other hand, 3358 of the 4718 mutants were observed to have abnormal morphology in at least one trait at FDR = 0.01 (Additional file 2: Table S1). Among these genes, 2241 were not detected as holistic morphological effectors (green in Fig. 2b, FDR = 0.01). Hereafter, these 2241 genes are defined as “specific morphological effectors,” which did not have significant holistic effects but affected at least one trait. Likewise, these mutants are defined as specific morphological mutants. The mutants of these specific morphological effectors displayed relatively weak but distinctive phenotypic changes. The other 1351 mutants had no detectable holistic or specific morphological alterations (black in Fig. 2b, FDR = 0.01). Because almost all mutants with holistic abnormality had at least one altered morphological trait (Fig. 2b), holistic morphological abnormality can be used as a more reliable index for yeast morphological mutants than specific abnormalities.

### Validation of holistic morphological effectors

We validated our identification of holistic morphological effectors by repeated phenotyping of 19 (1.7% of 1126) randomly selected deletion mutants with holistic morphological abnormalities and the same number of wild-type replicates (Additional file 3: Figure S2A). Haploid mutants in the non-essential gene deletion library were maintained for a large number of generations. Some gene-deletion mutants might exhibit increased mutation rates or gain unexpected mutations and thereby cause additional morphological phenotypes that are not associated with the targeted gene deletion mutation. Therefore, we started with heterozygous diploids, subjected them to sporulation and germination, and kept the number of generations between sporulation and fixation to a maximum of 50. We confirmed that most holistic morphological mutants exhibited significant gross morphological abnormalities (Additional file 3: Figure S2B, Additional file 4: Table S2). There was only one exception (*set5*Δ) out of the 19 strains tested, which is generally consistent with the intrinsic false discovery rate (FDR = 0.01). The deletion mutant with the greatest holistic morphological abnormalities among the tested strains was *cdc10*Δ, which was confirmed in the repeated experiment. Together, these data suggest that holistic morphological abnormality can be used as a reproducible index for yeast morphological mutants.

### Comparison of morphological phenotypes between holistic and specific mutants

**Fig. 3:**
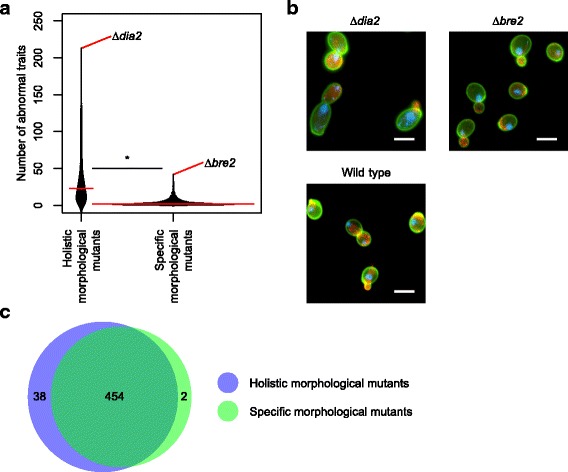
Comparison of morphological abnormality in holistic and specific morphological mutants. **a** Comparison of the number of abnormal phenotypes. Number of altered traits was counted for each mutant after detecting abnormal phenotypes at FDR = 0.01 (Additional file 2: Table S1). Asterisk indicates significant difference (*p* < 0.01 by Mann–Whitney *U* test). **b** Microscopic images of representative mutants of specific effectors and holistic effectors. The specific (*bre2*Δ) and holistic (*dia2*Δ) morphological mutants selected for Fig. 3a were extreme mutants. Scale bar indicates 5 μm. **c** Venn diagram showing overlap of altered traits between holistic and specific morphological mutants. The total number of altered traits in holistic and specific morphological mutants (FDR = 0.01) is shown

We compared the number of traits with abnormal phenotypes between holistic and specific morphological mutants (Additional file 2: Table S1). The number of traits with abnormal phenotypes for the holistic mutants was significantly higher than for the specific mutants (Fig. 3a, *p* < 0.01 by Mann–Whitney *U* test). The median numbers of altered traits in holistic and specific mutants were 23 (interquartile range [IQR]: 11 to 46) and 2 (IQR: 1 to 5), respectively, indicating that holistic mutants have more altered traits than specific mutants. The holistic mutant with the largest number of altered traits was *dia2*Δ, with abnormal phenotypes in 213 traits (Fig. 3a, b). Although the specific mutant with the largest number of altered traits (*bre2*Δ) had significantly abnormal phenotypes in 42 traits, it was indistinguishable from the wild-type control based on images of the cells (Fig. 3b), suggesting that the morphological phenotypes of specific mutants are often difficult to recognize without statistical analysis.

We compared the total number of altered traits between holistic and specific morphological mutants. Overall, 492 and 456 traits were detected in at least one holistic and specific morphological mutant, respectively, with 454 overlapping traits (Fig. 3c). More traits were used for phenotyping of holistic morphological mutants, likely due to the fact that holistic morphological mutants had greater impacts on morphology. These results suggest that the traits altered in holistic and specific morphological mutants remain similar in quantity but differ in quality in terms of the degree of abnormality.

### Association of fitness genes with holistic morphological effectors

**Fig. 4:**
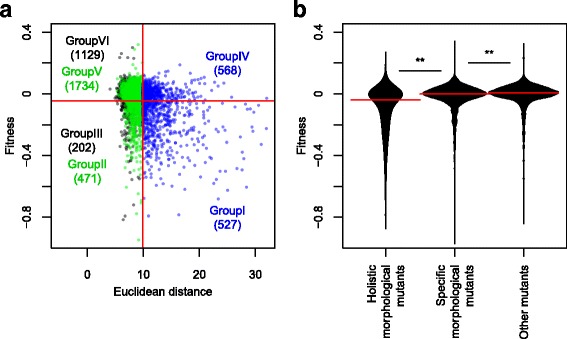
Relationship between holistic morphological abnormality and fitness. **a** Scatter plot of non-essential gene deletion mutants in terms of holistic morphological abnormality (x-axis) and fitness (y-axis) (Additional file 2: Table S1). Blue, green, and black circles indicate holistic morphological mutants, specific morphological mutants, and other mutants, respectively. Horizontal and vertical solid red lines indicate FDR = 0.01. Each number indicates the number of deletion mutants classified based on fitness and morphology. **b** Fitness in holistic morphological mutants, specific morphological mutants, and other mutants. Horizontal solid red lines indicate median values. ** indicates a significant difference at *p* < 0.01 by the Mann–Whitney *U* test

Previous studies have reported conflicting views on the association between fitness defects and morphological abnormalities. Perturbation of cell cycle progression results in Cdc phenotypes, which induce characteristic morphological phenotypes [24]. On the other hand, polarisome mutants display distinct morphological phenotypes, but no obvious growth defects [25]. To better understand holistic morphological effectors, we first comprehensively compared them with genes for fitness. We employed fitness data (Additional file 2: Table S1, Additional file 5: Figure S3) published previously [12], which revealed a weak but significant correlation between fitness defects and holistic morphological abnormalities (Spearman’s rank correlation coefficient, R = 0.25) (Fig. 4a). Holistic morphological mutants exhibited significantly slower growth than specific morphological mutants (*p* < 0.01 by Mann–Whitney *U* test) (Fig. 4b). Likewise, specific morphological mutants exhibited slower growth than other mutants (Fig. 4b). The fraction of strains exhibiting slow growth was significantly higher in holistic mutants than in others (Additional file 6: Figure S4). We also analyzed the relationships with other gene features compiled in Koch et al. [26] and found that several gene features are significantly correlated with holistic morphological abnormality (Additional file 7: Figure S5A) as well as fitness defect (Additional file 7: Figure S5B), but there was no detectable correlation with specific morphological abnormality (Additional file 7: Figure S5C). Because similar correlation patterns were observed for holistic morphological abnormality and fitness defect, we also analyzed the partial correlation coefficient between holistic morphological abnormality and each of the gene features that determine fitness. The results (Additional file 7: Figure S5D) showed that some gene features (expression level, codon adaptation index, co-expression degree, Nc, and copy number) had reduced correlations, while others (protein length) became more correlated. These data suggest that holistic morphological abnormality can be used to evaluate the importance of genes from a perspective other than fitness.

### Functional connectivity of holistic morphological effectors in a genome

**Fig. 5:**
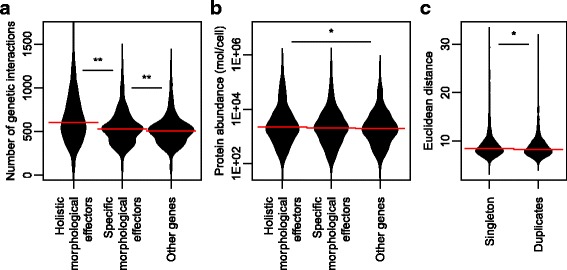
Comparison of holistic morphological effectors with other gene features. **a** The number of genetic interactions of holistic morphological effectors, specific morphological effectors, and others. **b** Protein expression levels of holistic morphological effectors, specific morphological effectors, and others. **c** Holistic morphological abnormality of singletons and duplicates. Horizontal solid red lines indicate median values. * and ** indicate significant differences at *p* < 0.05 and *p* < 0.01, respectively, as determined by the Mann–Whitney *U* test after Bonferroni correction

Genetic interaction networks are composed of a small number of highly connected nodes (hubs) and a large number of poorly connected nodes [16]. In these networks, deletion of a hub is more likely to result in strong phenotypic effects than deletion of other nodes. Therefore, we investigated whether holistic effectors are frequently observed as network hubs. Network hubs are defined here as genes with a large number of genetic interactions, as measured based on fitness [16] instead of morphology, because interaction data based on morphology are not currently available. We first analyzed the distribution of the number of genetic interactions among classes. Significantly more genetic interactions were observed in holistic effectors than in specific effectors (*p* < 0.01 by Mann–Whitney *U* test) (Fig. 5a). Likewise, more genetic interactions were observed in specific effectors than in other genes (Fig. 5a). We next analyzed the frequency of genetic interactions in each class. We found that the cumulative distribution function increased more slowly for holistic effectors than for other classes (Additional file 8: Figure S6A), implying that holistic effectors have more genetic interactions. In addition, the density of genetic interactions revealed that genes with more than 1783 interactions were usually holistic effectors (Additional file 8: Figure S6B). Taken together, our analysis indicated that holistic morphological effectors exhibit many genetic interactions, and thus act as intracellular network hubs.

### Holistic morphological effectors exhibit abundant protein expression

Because a gene exerts its functions through the protein expressed by the gene, we supposed that disruption of a gene without protein expression during vegetative growth would result in less severely affected phenotypes. To test this idea, we employed a comprehensive dataset of protein abundance in log-phase growing cells [27]. Comparing protein abundances among the holistic effectors, specific effectors, and other genes revealed a significant relationship between protein abundance and holistic effectors (Fig. 5b). The median protein abundance of holistic genes, specific genes, and other genes was 2.21 (IQR: 0.91 to 6.39), 2.06 (IQR: 0.75 to 5.37), and 1.92 (IQR: 0.79 to 4.67) thousand molecules per cell, respectively. Notably, protein abundance of the holistic gene was significantly higher than that of other genes among the three pairs (*p* < 0.05 by Mann–Whitney *U* test after Bonferroni correction) (Fig. 5b, asterisk). This finding suggests that deletion of a gene that does not encode an expressed protein results in less severe holistic morphological effects.

### Morphological phenotypes of deletion mutants for duplicate genes

Duplicated genes cause smaller fitness defects in yeast deletion mutants [18]. The frequency distribution of phenotype fitness for duplicate genes was significantly different from that for singletons. Because a weak correlation was observed between fitness defects and holistic morphological abnormalities, we aimed to determine whether this is also the case for morphological phenotypes. We compared holistic morphological abnormality in deletion mutants for duplicate genes and mutants of singlet genes. We employed 2507 duplicate and 1807 singlet genes described in Diss et al. [28] and found that deletion mutants for singletons resulted in greater morphological abnormality than those for duplicate genes (Fig. 5c). Duplicate genes include heteromer small-scale duplicates (SSDs), other SSDs, heteromer ohnologs, and other ohnologs. We compared these gene groups and found that singletons are significantly different from other ohnologs (*p* < 0.05, Mann-Whitney Utest) (Additional file 9: Figure S7). Taken together, our global analysis suggests that deleting a duplicate gene from the genome has little phenotypic effect on morphology.

### Functional categories of genes characterized by fitness and morphology

Based on fitness defects and holistic and specific morphological abnormalities, non-essential genes were classified into six groups (Fig. 4a), including holistic morphological effectors required for fitness (Group I, 527 genes), specific morphological effectors required for fitness (Group II, 471 genes), genes required only for fitness (Group III, 202 genes), holistic morphological effectors unnecessary for fitness (Group IV, 568 genes), specific morphological effectors unnecessary for fitness (Group V, 1734 genes), and genes not responsible for fitness or morphology (Group VI, 1129 genes). We performed gene ontology (GO) enrichment analysis with adjacent GO terms [29] (Additional file 10: Figure S8), and revealed that some gene functions are associated with each group except for Groups V and VI.

**Fig. 6:**
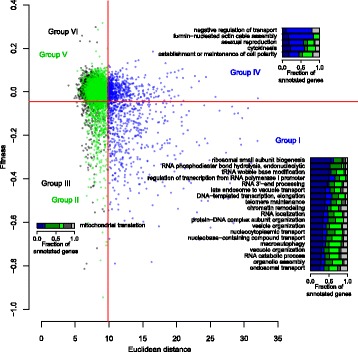
Functional enrichment in gene groups specified by fitness and morphology. Functional enrichment in gene groups I–VI. The scatter plot, colored dots, and solid red lines are as shown in Fig. 4a. Bar graphs associated with each group indicate the fractions of genes annotated with each adjacent gene ontology (GO) term. Bar colors: dark blue, dark green, dark gray, blue, green, and gray indicate gene groups I, II, III, IV, V, and VI, respectively

**Fig. 7:**
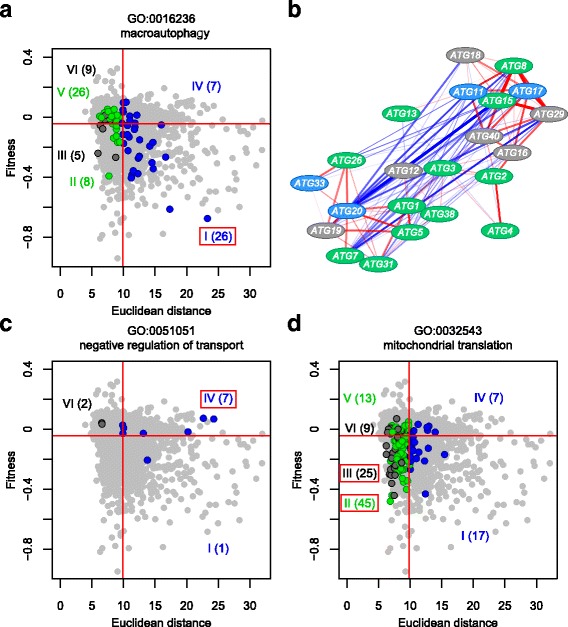
Distribution of deletion mutants for genes annotated to specific GOs. **a** Group I related to autophagy (GO:0016236). **b** Morphological similarity among autophagy-related gene deletion mutants. The subnetwork was described using Cytoscape (http://www.cytoscape.org/) and nodes were placed using the spring-embedded layout. Colors of nodes represent genes in group I and IV (blue), group II and V (green), group III and VI (gray) Red and blue edges indicate positive and negative correlations with morphological phenotype, respectively. Transparency of colors at edges is proportional to absolute R value. Wide, medium, and narrow edges indicate strong (0.6 < R value ≤0.8), moderate (0.4 < R value ≤0.6), and weak correlations (0.2 < R value ≤0.4), respectively. **c** Group IV related to negative regulation of transport (GO:0051051). **d** Groups II and III related to mitochondrial translation (GO:0032543). Blue, green, and dark gray circles indicate mutants of genes annotated with specific GOs. Gray circles indicate mutants of genes not annotated with the specified GOs. The number of annotated genes in each group is shown in parentheses. Red frames indicate gene groups related to a specific GO. The scatter plot, colored dots, and solid red lines are as shown in Fig. 4a

Statistical analysis indicated that defects in many essential biological processes, including ribosomal biogenesis, tRNA modification, RNA metabolism, vesicular transport, telomere maintenance, chromatin remodeling, nucleocytoplasm transport, autophagy, vacuole organization, organelle assembly, and endosomal transport, result in both fitness reduction and holistic morphological defects (FDR = 0.01 by Fisher’s exact test) (Group I in Fig. 6). Among gene functions related to Group I, we further analyzed autophagy (GO:0016236). Figure 7a shows that 26 out of 81 autophagy-related genes are enriched in Group I (*p* = 3.9 × 10^− 7^ by Fisher’s exact test). More than half of the mutated autophagy genes were either specific or holistic morphological effectors. It should be noted that many autophagy-related genes are activated upon starvation [30], although our morphological effectors were studied during vegetative growth. To determine whether the observed phenotypes are a consequence of autophagy alteration, we analyzed phenotypic similarity among the autophagy mutants. We noted morphological similarity among the *atg*Δ mutants (Fig. 7b), suggesting that the morphology of *atg*Δ mutants is not caused by unexpected off-target mutations or incidental experimental errors.

Among holistic effectors unnecessary for fitness (Group IV), genes annotated with the negative regulation of transport, polarisome, asexual reproduction, cytokinesis, and cell polarity terms are significantly enriched (FDR = 0.01 by Fisher’s exact test) (Fig. 6). As one example, 7 out of 10 genes involved in negative regulation of transport (GO:0051051) are enriched in Group IV (*p* = 3.5 × 10^− 5^ by Fisher’s exact test) (Fig. 7c), implying that these gene functions impact morphology rather than fitness. Among the genes required only for fitness (Group III), mitochondrial translation genes (GO:0032543) are significantly abundant in Group III (FDR = 0.01 by Fisher’s exact test) (Figs. 6, 7d). These genes are also observed significantly among specific effectors required for fitness (Group II) (Figs. 6, 7d), suggesting that their functions have more impact on fitness than on morphology.

**Fig. 8:**
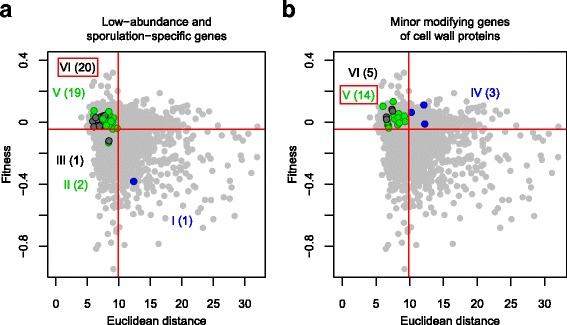
Enrichment of specific gene groups in Group V and VI. **a** Enrichment of low-abundance and sporulation-specific genes in Group VI. **b** Enrichment of minor cell wall protein-modifying genes in Group V. The symbols and colors used are as defined in Fig. 7

No adjacent GO terms were associated with Groups V and VI, and furthermore, the fractions of genes with no functional annotation were high in Group V and VI (Additional file 11: Figure S9). To identify hidden enriched gene groups, we manually selected a group of genes and tested their enrichment in these groups. For genes unrelated to fitness or morphology (Group VI), low-abundance and sporulation-specific genes were tested (Additional file 12: Table S3). Statistical analysis indicated that 20 out of 43 low-abundance and sporulation-specific genes are enriched among the genes unnecessary for fitness and morphology (Group VI) (*p* < 0.01 by Fisher’s exact test). These mutants generally exhibited no obvious fitness or morphological phenotypes under vegetative growth conditions, as expected (Fig. 8a). For specific effectors unnecessary for fitness (Group V), minor modification genes for cell wall proteins were selected (Additional file 13: Table S4), because many duplicate cell wall proteins play roles in cell morphology [31, 32], but not in fitness. Statistical analysis indicated that 14 out of 22 minor modification genes of cell wall proteins are enriched in specific effectors unnecessary for fitness (Group VI) (*p* < 0. 05 by Fisher’s exact test), as expected (Fig. 8b). Thus, our analysis clearly indicated that phenotypic strength in terms of fitness and morphology can be used to efficiently classify functional categories of genes.

## Discussion

Holistic and specific morphological effectors were comprehensively investigated in the budding yeast *Saccharomyces cerevisiae*. We identified 1126 (24%) holistic morphological effectors that cause severe gross morphological abnormality when deleted. These holistic morphological effectors are indispensable genes in morphogenesis. We also identified 2241 (47%) specific morphological effectors that did not affect morphology to a great extent, but that significantly influenced yeast morphology in specific ways. These specific morphological effectors are also important in yeast morphogenesis, but less so than holistic effectors. An association study revealed that 527 holistic morphological effectors overlap with fitness genes. Given that different functional categories of genes are associated with fitness and holistic morphological effectors, holistic morphological abnormalities can be used for many purposes.

### Morphological profiling and holistic morphological abnormality

High-dimensional morphological data contains a signature profile (morphological profile) and holistic morphological abnormality information. The morphological profile has been widely used [20] to detect a close relationship between the morphological phenotype and functional annotation of a gene [21], morphological similarity between mutants and chemicals for drug target prediction [33–35], and clustering of mutants with similar morphology [36, 37]. On the other hand, holistic morphological abnormalities have rarely been used to characterize deletion mutants. Such abnormality (total effect size in morphology) was compared with the degree of pleiotropy in an evolutionary genetics study [23]. In many other comprehensive analyses on yeast morphology [10, 22, 38–40], only specific abnormalities were defined and described in each mutant. Thus, we propose here that in addition to the morphological profile, holistic morphological abnormality of a gene deletion mutant can be used as a gene feature in high-dimensional phenotyping studies.

### Validation of holistic morphological effectors

Calculation of holistic morphological abnormality was carried out in three steps, including normalization of 501-dimensional morphological data using a generalized linear model, extraction of independent and stable morphological features using PCA, and calculation of the Euclidean distance from the mean value of the wild type. We used Euclidean distance rather than Maharanobis distance due to its robustness against experimental errors. Because most (99.2%) of the non-essential deletion mutants with holistic morphological abnormality also had at least one significantly altered trait, holistic morphological abnormality can be used as a reliable indicator for yeast morphology. We validated the reproducibility of holistic morphological effectors by repeating the experiments. Our results indicated that the holistic morphological abnormalities of the deletion mutants were mostly due to on-target gene deletion mutations. Thus, holistic morphological abnormality is a reliable and reproducible index of morphological abnormality.

### Relationship between fitness genes and holistic morphological effectors

Yeast genes that contribute significantly to phenotypic strength have been thoroughly studied in terms of fitness. We revealed that holistic morphological effectors overlap with fitness genes, and there was a significant correlation between these two gene features. Like fitness genes [13, 26], holistic morphological effectors were associated with the degree of genetic interaction and the abundance of expressed proteins. However, it should be noted that genes for fitness and morphology were not identical. The apparent reason for the discrepancy is that there are likely phenotypic traits that affect fitness but are not related to morphology. In addition, different morphological traits may have different degrees of correlation with fitness, such that larger holistic morphological abnormalities do not necessarily have larger fitness effects. Thus, functional categories of genes can be classified based on fitness and holistic morphological degree. We also revealed that singleton genes affect both fitness [18] and morphology more strongly than duplicate genes. Although the deletion of heteromer SSDs reduced fitness more than deletion of heteromer ohnologs, there were no holistic morphological differences. This may be explained by the biased distribution of SSDs in gene functions. For example, many genes encoding cell wall proteins are duplicate genes, causing changes only in morphology but not in fitness when deleted. Combining fitness and morphological phenotypes will contribute to a better understanding of gene functions and cellular networks.

### Gene functions specifically related to morphology

We revealed that many non-essential genes are more important to morphology than to fitness. Since Group IV and Group V genes impact morphology more than fitness, the main functions of these genes are assigned to cell morphogenesis. Among the functions of the genes enriched in Group IV and Group V, polarisome [25], asexual reproduction, cytokinesis [41], cell polarity [42], and cell wall proteins [31, 32] are known to be involved in cell morphogenesis. Genes encoding factors for negative regulation of transport were unexpectedly enriched, which may suggest an unknown link between the transport of small metabolites and cell morphogenesis. Since a large number (2302) of non-essential genes belong to Group IV and Group V, further study of these genes will uncover the molecular mechanism as well as the cellular network involved in cell morphogenesis.

### Function of autophagy-related genes in morphology

Many autophagy-related genes are expressed under starvation conditions [30]. Because morphology was observed in early log phase cells [21], most autophagy-related mutants are unlikely to exhibit obvious morphological changes. However, many holistic and specific morphological effectors exist among autophagy-related genes. High morphological similarity between autophagy-related deletion mutants suggested that the observed phenotype is due to autophagy inhibition. Atg17 and Atg29 function together in starvation-induced non-selective autophagy [43–45]. A recent study indicated that the Atg17-Atg29 complex interacts with Atg11 [46]. Morphological similarity among Atg11, Atg17, and Atg29 mutants implied that they might play similar roles during vegetative growth. It should be noted that the morphological phenotypes of Atg17, Atg29, and Atg15 are anti-correlated with Atg20, which is involved in the cytoplasm-vacuole targeting (Cvt) pathway [47]. This result strongly suggested that Atg17, Atg29, and Atg15 play other roles during vegetative growth in addition to the Cvt pathway. Because either holistic or specific morphological effectors appeared in 71% of non-essential gene mutants, unforeseen morphological abnormalities are likely associated with other gene deletion mutants.

## Conclusions

This is the first genome-scale analysis to define and characterize holistic morphological effectors, which are defined as non-essential genes that have significant holistic effects on morphology. We propose that holistic morphological abnormality is a useful index for the study of gene function. It is independent of morphological signature, but reflects the total degree of the morphological effect. The simplest application of non-essential gene deletion mutants is to determine the importance of a gene in morphology. Because holistic morphological abnormality has a weak correlation with fitness, it can provide another ruler for measuring gene importance. Second, holistic morphological abnormality can be used for classification of gene functions. As shown in this study, the combination of fitness and holistic morphological abnormality enables classification of gene function. Therefore, a two-dimensional plot of fitness and holistic morphological abnormality provided a powerful tool for the characterization of non-essential genes. Third, holistic morphological abnormality can be used to interpret morphological similarity. Loss of morphological similarity is explained by either actual dissimilarity of the profile or weak morphological abnormality. Information on weak holistic morphological abnormality may be useful in understanding the absence of morphological similarity. Alternatively, morphological comparison using only holistic morphological effectors may be more reliable. Finally, holistic morphological abnormality can be used to better understand other gene features and gene networks. Holistic morphological abnormality is correlated with many genetic and other features and therefore can be used to integrate this information. Holistic morphological abnormality can also be defined in relation to any perturbation, such as gene or allele deletion, drug treatment, or environmental change. Development of further applications for holistic morphological abnormality is expected in the future in functional genomics as well as cell biology and evolutionary genetics.

## Methods

### Strains and original morphological dataset

#### Morphological dataset of 4718 non-essential gene mutants and wild-type replicates

Morphological data of 4718 non-essential gene deletion mutants and 109 wild-type replicates were obtained by subjecting microscopic images of yeast cells to the image processing program CalMorph (ver. 1.2) as previously described [21, 48]. Selected replicated data (*n* = 109) of *his3*/*yor202w*Δ were used as wild-type data.

#### Morphological dataset of 19 non-essential gene mutants and wild-type replicates during validation analysis

To obtain fresh haploid gene-deletion mutants, we used heterozygous diploids for 19 non-essential genes purchased from EUROSCARF (Frankfurt, German) (Additional file 4: Table S2). Diploid cells were freshly grown on YPD agar plates (2% dextrose, 2% peptone, 1% yeast extract, and 2% agar), then patched onto GNA pre-sporulation agar plates (5% dextrose, 3% nutrient broth, 1% yeast extract, and 2% Bacto agar), and grown at 25 °C for 1 day. Colonies were transferred into 2 mL of sporulation medium (10% potassium acetate, 0.005% zinc acetate, +Ura + His +Leu) and cultured at 25 °C for 6 days. Tetrads were dissected with a Tetrad Dissection Microscope (Singer Instruments) after 5 min of treatment with 1 mg/mL zymolyase, then grown on YPD agar plates with or without 1 M sorbitol until ~ 2 mm size colonies appeared (~ 20 generations, no more than 3 days). We prepared frozen stocks of single colonies at this point for later experimental use. Cell stocks were struck out on YPD plates and grown at 25 °C for a maximum of ~ 48 h (~ 20 generations). Morphological data for the deletion strains were acquired as described previously [21], taking an additional ~ 10 generations. In total, the number of cell generations during spore germination and fixation was kept to a maximum of 50.

### Data processing

#### Noise phenotypes

Coefficient of variation values were highly dependent on the mean values in a non-linear manner [49], and therefore were not suited for normalization. Instead, we defined noise values as the residuals between observed and predicted values, as described previously [50].

#### Detection of specific morphologically abnormal mutants

The probability distribution of the wild type for each trait of the 501 parameters was estimated using maximum likelihood estimation (MLE) with one of four probability density functions (gamma, beta, Gaussian, and beta-binomial distribution), as described previously [48]. We then mapped every non-essential deletion mutant, calculated its *p* value as morphological abnormality from the wild type for each trait (two-sided one-sample test), and identified the lowest *p* value among 501 traits as the “specific morphological abnormality” (Additional file 2: Table S1). MLE and calculation of the *p* value were performed using the gamlss function in R software’s (http://www.r-project.org) gamlss package [51]. The FDR, a rate of type I errors in the rejected null hypothesis due to multiple comparisons, was calculated using the qvalue R function in the qvalue package [52].

#### Normalization of morphological data

Morphological data for mutants and wild-type replicates were normalized by transformation into the Z value of the Wald test based on the mean and dispersion estimated via MLE for the wild type (*n* = 109) using the coeftest R function in the lmtest package [53].

#### Dimensional reduction with PCA

PCA was performed using the Z values of 4718 deletion mutants with the prcomp R function in the stats package. The degenerate orthogonal space does not contain intrinsic correlations between the morphological parameters or between the mutants. The first 19, 32, 57, and 108 PCs reached 60, 70, 80, and 90% of the CCR, respectively (Additional file 1: Figure S1B). PC scores for the wild type were calculated by projecting Z values of the wild type onto the PC axes.

#### Calculation of Euclidean distances

Euclidean distance (Fig. 1b) was calculated as the square root of the sum-of-squares of standardized PC scores. PC scores of the mutants and wild type for 57 PCs (reaching 80% of the CCR) were standardized by the mean and variance of PC scores of the wild type. For a 57-dimensional space, the distance *d* of *i*th mutant was calculated with the following equation:

$$ d\left({p}_i,\kern0.5em q\right)=\sqrt{\sum_{j=1}^{57}{\left({p}_{ij}-\widehat{q_j}\right)}^2}, $$

where *p*_*ij*_ and $\widehat{q_j}$ are the *j*th PC score of the *i*th mutant and the mean of the *j*th PC score of the wild type (Additional file 2: Table S1).

#### Detection of holistic morphologically abnormal mutants

The holistic abnormality of each mutant was estimated as its Euclidean distance from the center of 109 replicates of the wild type in 57-dimensional orthogonal space. The Euclidean distances of the 4718 non-essential gene deletion mutants were compared with the distribution of 109 wild-type replicates. We calculated the false discovery rate (FDR = 0.01) by fitting a gamma distribution to the distribution of the wild type using the gamlss function in the R package gamlss [51]. Holistic morphological abnormal mutants were identified as those with Euclidean distances larger than FDR = 0.01 (right side of the vertical red line in Fig. 2a).

### Analysis of gene features

#### Genetic interactions

The number of genetic interactions was counted for 5549 open reading frames for which significant genetic interactions (*ε* score) were detected at *p* < 0.05 (lenient cutoff) from pair-wise interaction of raw genetic interaction datasets [16]. To standardize the number of genetic interactions, we divided the number of significant interactions counted in each pair-wise comparison by the number of experiments.

#### Fitness

To estimate fitness, we employed a previously published dataset of logarithmic strain growth rate coefficients for haploid non-essential gene deletion mutants grown on basal medium (LSC_basal_) [12]. We calculated the *p* value as the significance of lower fitness from the wild type of each strain based on one tail of the estimated probability distribution [12] using the pnorm function in the stats package of R. FDR was estimated using the qvalue R function in the qvalue package [52] (Additional file 2: Table S1).

#### Functional enrichment analysis

To determine the significance of enriched GO terms, Fisher’s exact test was performed using the fisher.test function in the R stats package. The FDR was calculated using the qvalue function of the R qvalue package [52]. We summarized the long list of enriched GO terms (FDR = 0.01) by removing redundant terms using the web-based program REVIGO [29] with the following options: similarity cutoff = 0.5, database for GO term sizes = “*Saccharomyces cerevisiae*”, semantic similarity measure = “Lin” [54] (Additional file 10: Figure S8).

#### Calculation of morphological similarity

Calculation of morphological similarity was performed as previously described [33]. Briefly, the Z values of the wild type were subjected to PCA using the prcomp function in the stats package of R. PC scores of mutants were then calculated by projecting the Z values of mutants onto PC axes. Pearson’s product moment correlation coefficient (morphological similarity) between mutants was calculated from the PC scores of the first 95 PCs (99% of the CCR) using the cor function in the R stats package.

## Additional files

Additional file 1: **Figure S1.** Dimensional reduction of morphological data through principal component analysis (PCA). (A) Cumulative contribution ratio (CCR) of PCA based on data of 4718 gene deletion mutants. Black bars indicate the contribution ratio of each PC (left axis). Red circles and curve indicate CCR (right axis). Horizontal and vertical red dashed lines indicate CCR = 0.8 and PC57, respectively. (B) Variance of PC scores in each PC. Black and yellow bars indicate 4718 gene deletion mutants and the wild type, respectively. Vertical red lines indicate the position of PCs reaching the indicated CCR. (C) Proportion of the number of PCs of wild type with larger variance than that of deletion mutants. Proportion of the number of PCs was counted for each indicated range of the CCR. (PDF 284 kb)

Additional file 2: **Table S1.** Holistic morphological abnormalities, specific morphological abnormalities, and fitness defects of non-essential gene mutants. (XLS 1168 kb)

Additional file 3: **Figure S2.** Validation of morphological phenotypes of holistic morphological mutants. (A) Nineteen randomly selected deletion mutants with holistic morphological abnormalities. Scatter plot of non-essential gene deletion mutants in holistic morphological abnormality (x-axis) and specific morphological abnormality (y-axis). Cyan, gray, and orange circles indicate the 19 selected mutants, other mutants, and 109 replicates of the wild type. Horizontal and vertical red lines indicate FDR = 0.01. (B) Confirmation of holistic morphological abnormality. Holistic morphological abnormality of each mutant was estimated by the Euclidean distance from the mean of 19 replicates of the wild type in 57-dimensional orthogonal space. Red and blue boxes indicate mutants (left axis) and 19 replicates of the wild type (right axis), respectively. Vertical solid red line indicates FDR = 0.01. Blue curved line indicates a gamma distribution fitted to the wild type. (PDF 3266 kb)

Additional file 4: **Table S2.** Holistic morphological abnormalities and genotypes of 19 non-essential gene mutants during validation analysis. (XLS 35 kb)

Additional file 5: **Figure S3.** Distribution of non-essential deletion mutants with fitness defects. Dark gray and light gray boxes indicate mutants of non-essential genes with significantly slower growth and normal growth, respectively (left axis). Vertical solid red line indicates FDR = 0.01. Blue curved line indicates normal distribution fitted to the wild type (right axis). (PDF 117 kb)

Additional file 6: **Figure S4.** Proportion of holistic and specific morphological mutants among mutants with and without growth defects. Right and left bar graphs indicate fractions of morphological phenotypes in mutants with slow and normal growth, respectively. Blue, green, and black bars indicate holistic morphological mutants, specific morphological mutants, and other mutants, respectively. The fraction of holistic morphological mutants was significantly higher in mutants with slow growth than in mutants with normal growth (*p* < 0.01 by Fisher’s exact test). (PDF 99 kb)

Additional file 7: **Figure S5.** Relationships with gene features. (A) Pearson’s product-moment correlation coefficients between holistic morphological abnormality and each gene feature. (B) Pearson’s product-moment correlation coefficients between fitness defect and each gene feature. (C) Pearson’s product-moment correlation coefficients between specific morphological abnormality and each gene feature. (D) Partial correlation coefficients between holistic morphological abnormality and each gene feature that controls fitness. Details of the gene features were previously described [26]. ** and * indicate *p* < 0.01 and *p* < 0.05, respectively, when testing for no correlation. Error bars indicate 95% confidential intervals. (PDF 8 kb)

Additional file 8: **Figure S6.** Distribution of genetic interactions. (A) Empirical cumulative distribution of the number of genetic interactions for each gene group. Blue, green, and black points indicate holistic morphological effectors, specific morphological effectors, and others, respectively. (B) Proportion of density of number of genetic interaction in each gene group described in a 100% stacked area chart. Blue, green, and black areas indicate holistic morphological effectors, specific morphological effectors, and others. The proportion for each number of genetic interactions was calculated from the density of genetic interactions for the corresponding gene. (PDF 1580 kb)

Additional file 9: **Figure S7.** Comparison of holistic morphological abnormality among singletons and duplicates of various types. Holistic morphological abnormality of singletons, heteromer small-scale duplicates (SSDs), other SSDs, heteromer ohnologs, and other ohnologs. Horizontal solid red lines indicate median values. Asterisk indicates a significant difference (*p* < 0.05 based on the Mann–Whitney *U* test after Bonferroni correction). (PDF 195 kb)

Additional file 10: **Figure S8.** Representation of gene functions with adjacent GO terms. Gray and black bars indicate the number of GO terms after summarization with REVIGO [29] using similarity cutoffs of 0.5 and 0.7, respectively (left y-axis). Four semantic similarity measures are supported by REVIGO: Resnik’s, Lin’s, Jiang and Conrath’s measures, and the SimRel measure [54]. Red dashed line indicates the number of GO terms prior to summarization. Right y-axis indicates the fraction of GO terms (after summarization/before summarization). Orange frame indicates the condition eventually selected. (PDF 108 kb)

Additional file 11: **Figure S9.** Fractions of genes with unknown function. Each bar indicates the fraction of genes with unknown functions identified by direct annotation to GO:0008150 (biological process) in each gene group (I–VI). (PDF 112 kb)

Additional file 12: **Table S3.** List of low-abundance and sporulation-specific genes used in this study. (XLS 32 kb)

Additional file 13: **Table S4.** List of minor modification genes for cell wall proteins used in this study. (XLS 29 kb)
