# Supplementary figures and images for: Global study of holistic morphological effectors in the budding yeast Saccharomyces cerevisiae
Source: BMC Genomics. 2018 Feb 20;19:149. doi: 10.1186/s12864-018-4526-z (PMC5819264; doi:10.1186/s12864-018-4526-z)

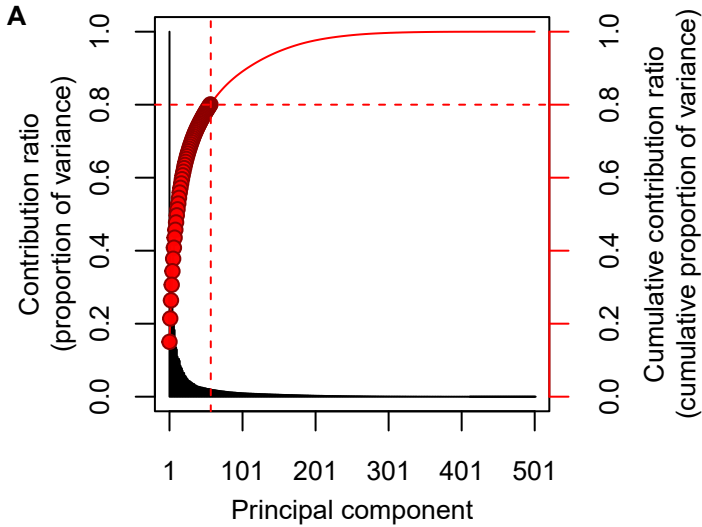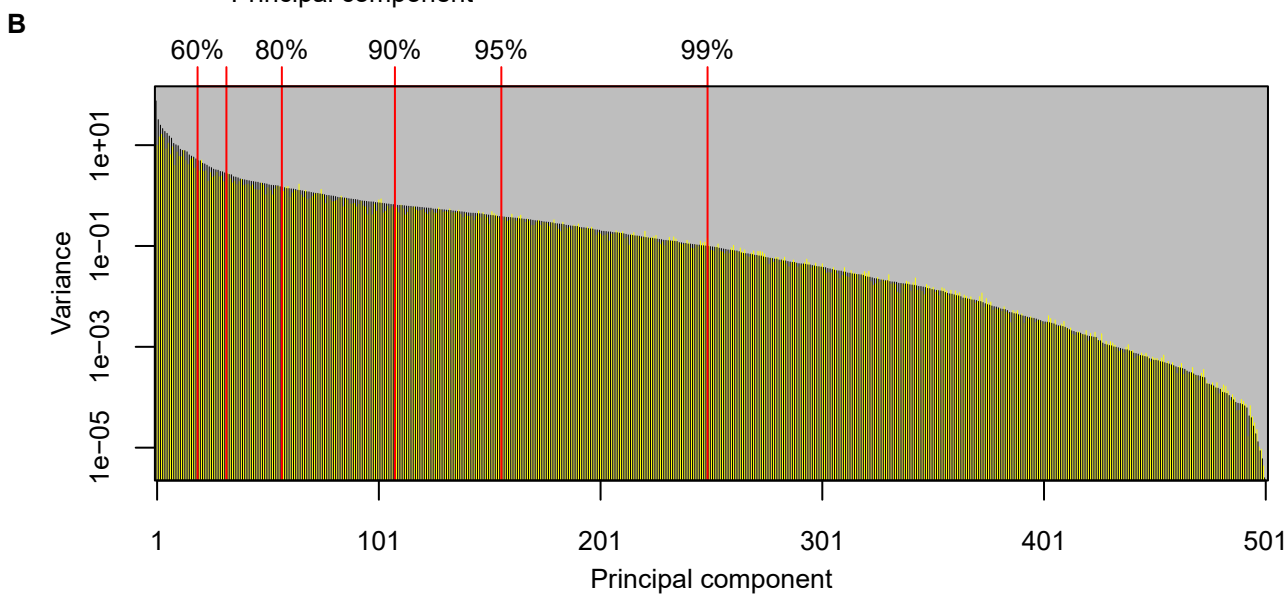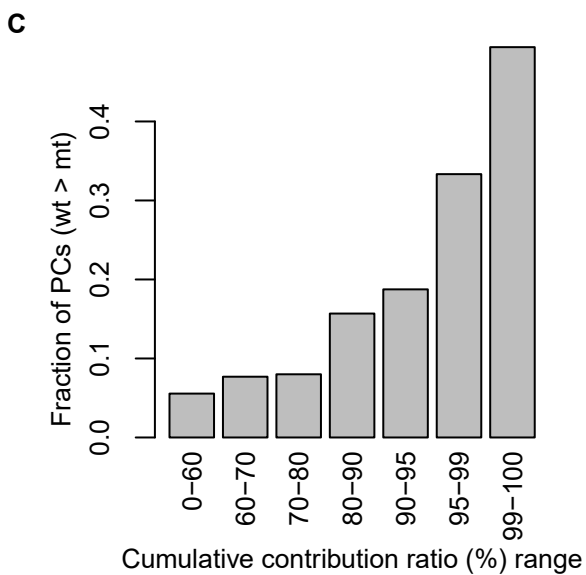

Supplement: Supplementary file 1 — Figure S1. Dimensional reduction of morphological data through principal component analysis (PCA). (A) Cumulative contribution ratio (CCR) of PCA based on data of 4718 gene deletion mutants. Black bars indicate the contribution ratio of each PC (left axis). Red circles and curve indicate CCR (right axis). Horizontal and vertical red dashed lines indicate CCR = 0.8 and PC57, respectively. (B) Variance of PC scores in each PC. Black and yellow bars indicate 4718 gene deletion mutants and the wild type, respectively. Vertical red lines indicate the position of PCs reaching the indicated CCR. (C) Proportion of the number of PCs of wild type with larger variance than that of deletion mutants. Proportion of the number of PCs was counted for each indicated range of the CCR. (PDF 284 kb) [file 12864_2018_4526_MOESM1_ESM.pdf]

**A**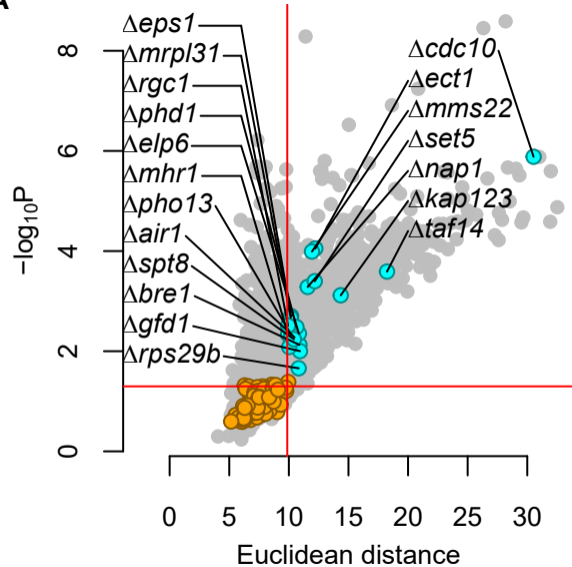**B**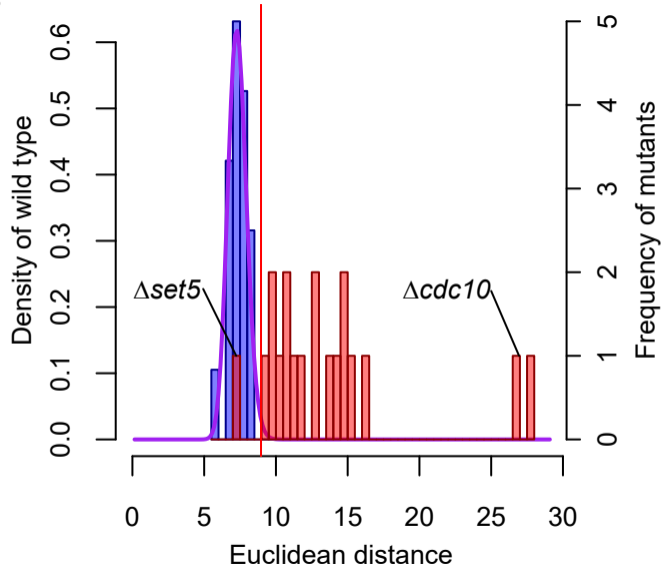

Supplement: Supplementary file 3 — Figure S2. Validation of morphological phenotypes of holistic morphological mutants. (A) Nineteen randomly selected deletion mutants with holistic morphological abnormalities. Scatter plot of non-essential gene deletion mutants in holistic morphological abnormality (x-axis) and specific morphological abnormality (y-axis). Cyan, gray, and orange circles indicate the 19 selected mutants, other mutants, and 109 replicates of the wild type. Horizontal and vertical red lines indicate FDR = 0.01. (B) Confirmation of holistic morphological abnormality. Holistic morphological abnormality of each mutant was estimated by the Euclidean distance from the mean of 19 replicates of the wild type in 57-dimensional orthogonal space. Red and blue boxes indicate mutants (left axis) and 19 replicates of the wild type (right axis), respectively. Vertical solid red line indicates FDR = 0.01. Blue curved line indicates a gamma distribution fitted to the wild type. (PDF 3266 kb) [file 12864_2018_4526_MOESM3_ESM.pdf]

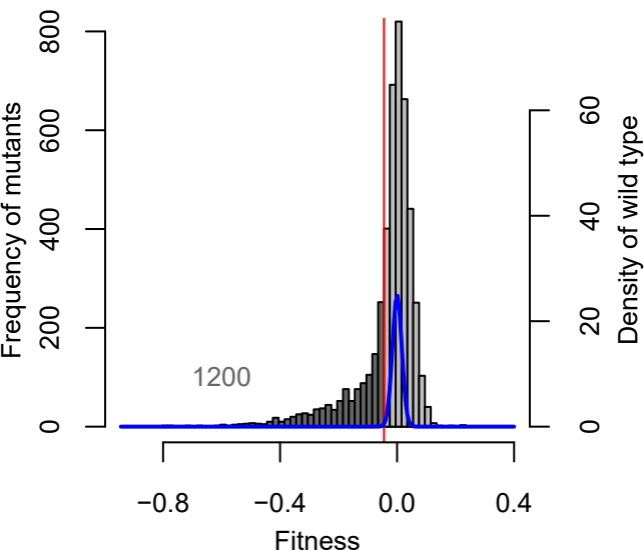

Supplement: Supplementary file 5 — Figure S3. Distribution of non-essential deletion mutants with fitness defects. Dark gray and light gray boxes indicate mutants of non-essential genes with significantly slower growth and normal growth, respectively (left axis). Vertical solid red line indicates FDR = 0.01. Blue curved line indicates normal distribution fitted to the wild type (right axis). (PDF 117 kb) [file 12864_2018_4526_MOESM5_ESM.pdf]

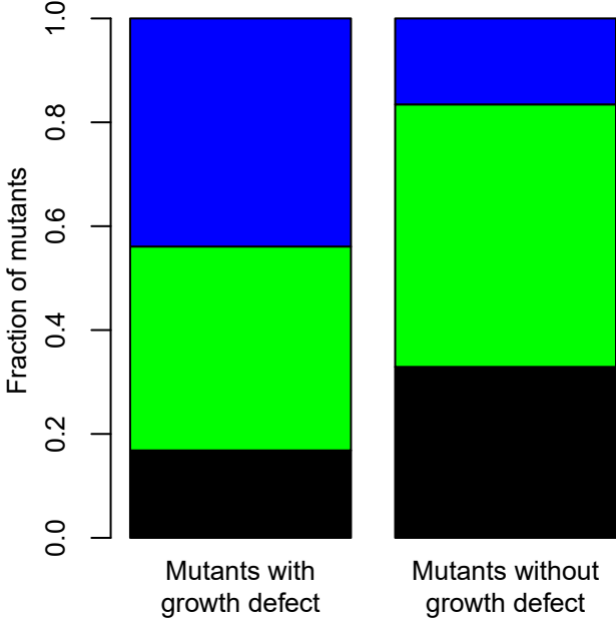

Supplement: Supplementary file 6 — Figure S4. Proportion of holistic and specific morphological mutants among mutants with and without growth defects. Right and left bar graphs indicate fractions of morphological phenotypes in mutants with slow and normal growth, respectively. Blue, green, and black bars indicate holistic morphological mutants, specific morphological mutants, and other mutants, respectively. The fraction of holistic morphological mutants was significantly higher in mutants with slow growth than in mutants with normal growth (p < 0.01 by Fisher’s exact test). (PDF 99 kb) [file 12864_2018_4526_MOESM6_ESM.pdf]

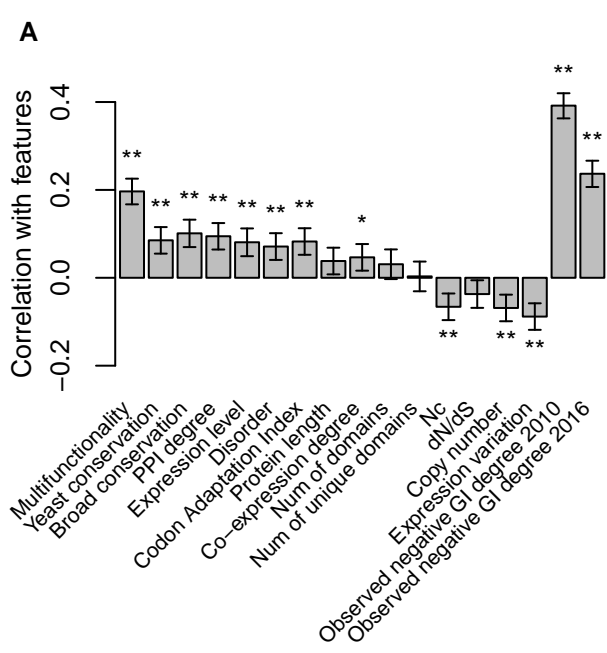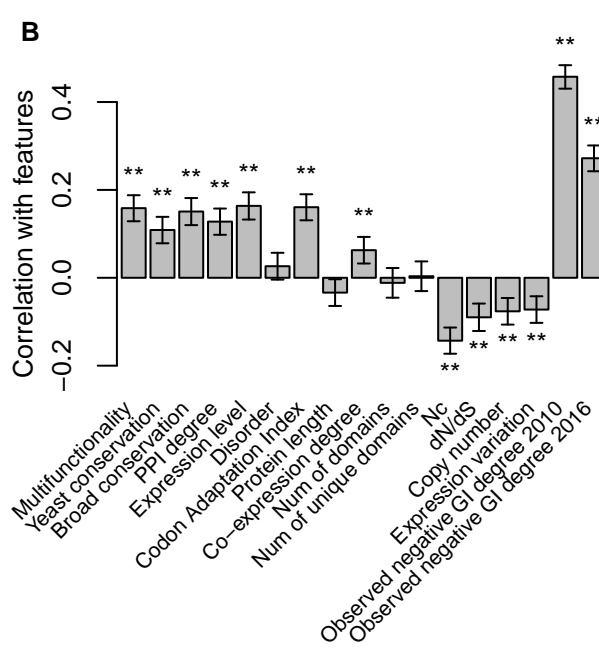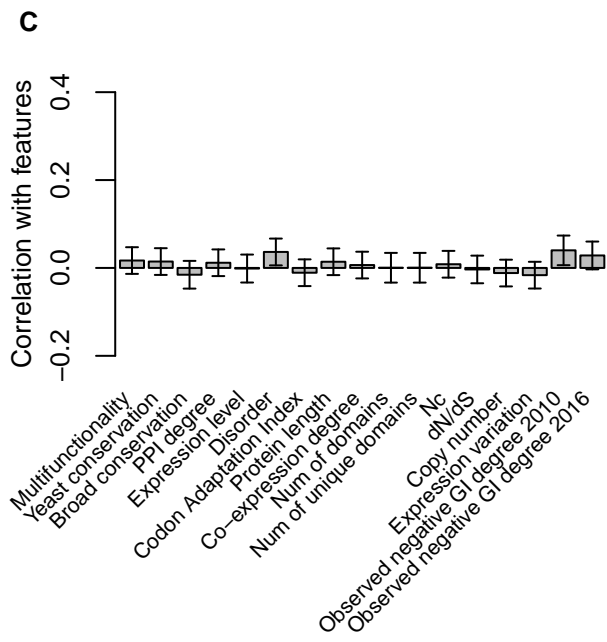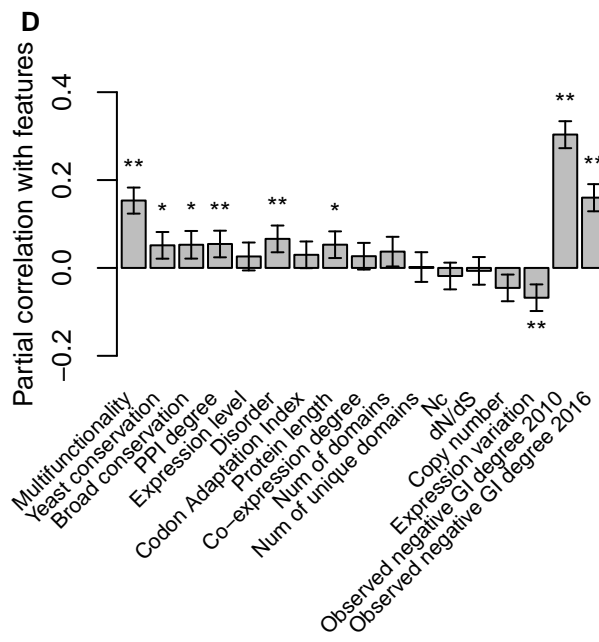

Supplement: Supplementary file 7 — Figure S5. Relationships with gene features. (A) Pearson’s product-moment correlation coefficients between holistic morphological abnormality and each gene feature. (B) Pearson’s product-moment correlation coefficients between fitness defect and each gene feature. (C) Pearson’s product-moment correlation coefficients between specific morphological abnormality and each gene feature. (D) Partial correlation coefficients between holistic morphological abnormality and each gene feature that controls fitness. Details of the gene features were previously described [26]. ** and * indicate p < 0.01 and p < 0.05, respectively, when testing for no correlation. Error bars indicate 95% confidential intervals. (PDF 8 kb) [file 12864_2018_4526_MOESM7_ESM.pdf]

**A**

Cumulative distribution

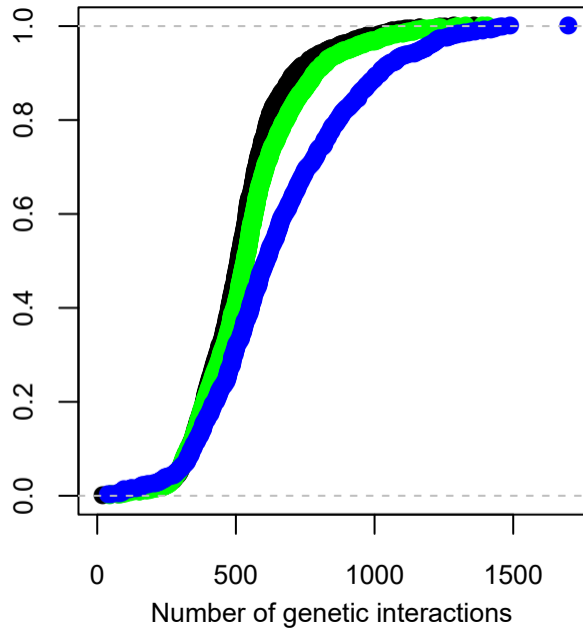**B**

Proportion of density

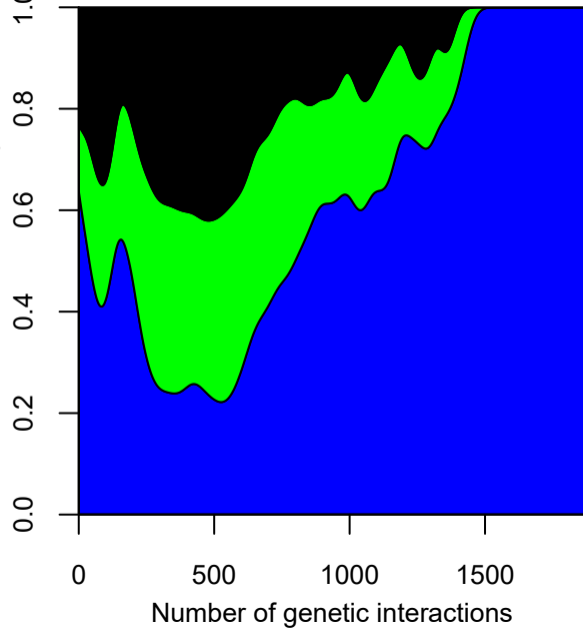

Supplement: Supplementary file 8 — Figure S6. Distribution of genetic interactions. (A) Empirical cumulative distribution of the number of genetic interactions for each gene group. Blue, green, and black points indicate holistic morphological effectors, specific morphological effectors, and others, respectively. (B) Proportion of density of number of genetic interaction in each gene group described in a 100% stacked area chart. Blue, green, and black areas indicate holistic morphological effectors, specific morphological effectors, and others. The proportion for each number of genetic interactions was calculated from the density of genetic interactions for the corresponding gene. (PDF 1580 kb) [file 12864_2018_4526_MOESM8_ESM.pdf]

Euclidean distance

5 10 15 20 25 30

Other  
SSDs

Heteromer  
SSDs

Singletons

Heteromer  
ohnologs

Other  
ohnologs

\*

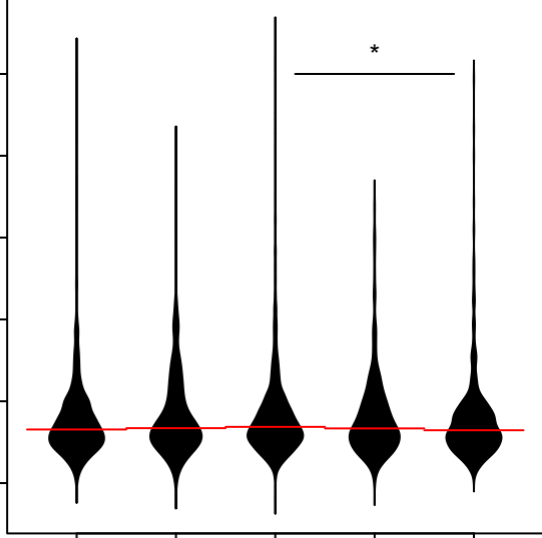

Supplement: Supplementary file 9 — Figure S7. Comparison of holistic morphological abnormality among singletons and duplicates of various types. Holistic morphological abnormality of singletons, heteromer small-scale duplicates (SSDs), other SSDs, heteromer ohnologs, and other ohnologs. Horizontal solid red lines indicate median values. Asterisk indicates a significant difference (p < 0.05 based on the Mann–Whitney U test after Bonferroni correction). (PDF 195 kb) [file 12864_2018_4526_MOESM9_ESM.pdf]

Number of GO terms

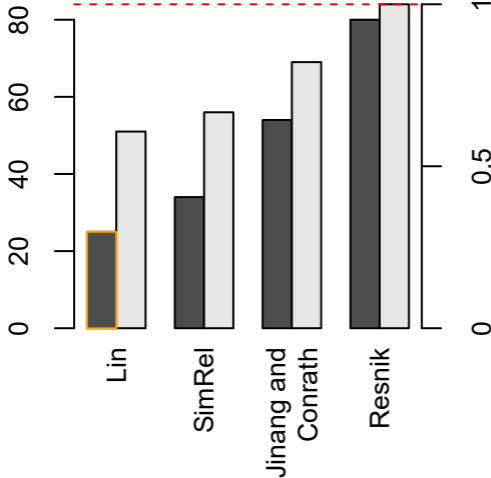

Fraction of GO terms  
(After summarization  
/ Before summarization)

Supplement: Supplementary file 10 — Figure S8. Representation of gene functions with adjacent GO terms. Gray and black bars indicate the number of GO terms after summarization with REVIGO [29] using similarity cutoffs of 0.5 and 0.7, respectively (left y-axis). Four semantic similarity measures are supported by REVIGO: Resnik’s, Lin’s, Jiang and Conrath’s measures, and the SimRel measure [54]. Red dashed line indicates the number of GO terms prior to summarization. Right y-axis indicates the fraction of GO terms (after summarization/before summarization). Orange frame indicates the condition eventually selected. (PDF 108 kb) [file 12864_2018_4526_MOESM10_ESM.pdf]
