# Supplementary material for: Global study of holistic morphological effectors in the budding yeast Saccharomyces cerevisiae
Source: BMC Genomics. 2018 Feb 20;19:149. doi: 10.1186/s12864-018-4526-z (PMC5819264; doi:10.1186/s12864-018-4526-z)
Supplement: Supplementary file 11 — Figure S9. Fractions of genes with unknown function. Each bar indicates the fraction of genes with unknown functions identified by direct annotation to GO:0008150 (biological process) in each gene group (I–VI). (PDF 112 kb) [file 12864_2018_4526_MOESM11_ESM.pdf]

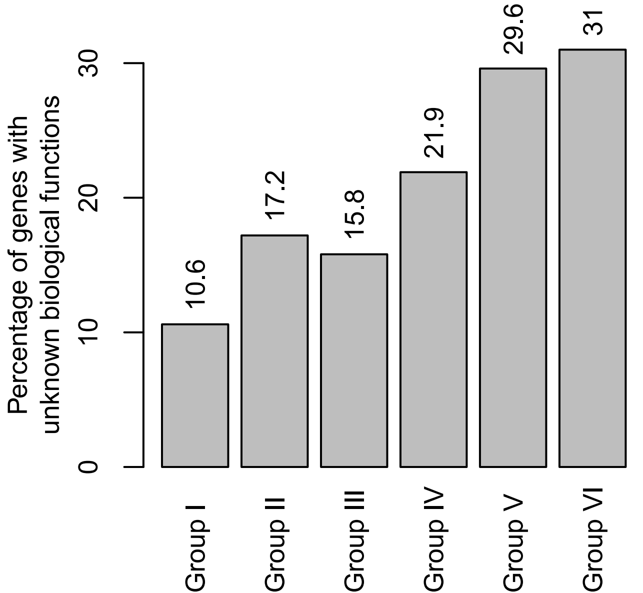
Percentage of genes with
unknown biological functions
0
10
20
30
10.6
17.2
15.8
21.9
29.6
31
Group I
Group II
Group III
Group IV
Group V
Group VI
